# Artificial Intelligence in Trauma and Orthopaedic Surgery: A Comprehensive Review From Diagnosis to Rehabilitation

**DOI:** 10.7759/cureus.92280

**Published:** 2025-09-14

**Authors:** Ahmed Mohamed, Alaa Elasad, Usman Fuad, Ioannis Pengas, Adham Elsayed, Prabhakar Bhamidipati, Peter Salib

**Affiliations:** 1 Trauma and Orthopaedics, Royal Cornwall Hospital, Truro, GBR; 2 General Practice, Zagazig University, Zagazig, EGY; 3 Emergency Medicine, Norfolk and Norwich University Hospitals NHS Foundation Trust, Norwich, GBR

**Keywords:** artificial intelligence, computer-assisted surgery, deep learning, machine learning, orthopaedic surgery, predictive analytics, trauma surgery

## Abstract

Artificial intelligence (AI) has presented clinical maturity in healthcare applications. AI is reshaping orthopaedic practice by enhancing the speed and efficiency of clinical decision-making, surgical planning, and research workflows. AI enables clinicians to optimize the care pathway through rapid data processing, pattern recognition, and predictive modelling. This review examines the current AI applications across the entire spectrum of orthopaedic care and its contribution to patient care and resource utilization. Despite these promising developments, several barriers prevent widespread adoption, including concerns regarding algorithm transparency, data privacy, potential bias in training datasets, and implementation costs. The path forward requires the development of explainable AI systems that clinicians can trust and validate. As AI technology continues to evolve, success will depend on augmenting human judgment with machine precision to deliver optimal care for patients with musculoskeletal conditions.

## Introduction and background

Artificial intelligence (AI) has been extensively used in medicine. Trauma and orthopaedics provide a fertile ground for AI applications [1]. Globally, the burden of musculoskeletal conditions continues to rise, with trauma remaining the leading cause of disability and death [2]. Simultaneously, the ageing population in developed countries has increased the incidence of degenerative joint diseases and fragility fractures [3]. This growing demand for orthopaedic services, combined with the drive for better outcomes and greater efficiency, has accelerated the adoption of AI tools that can enhance clinical decision-making and improve the delivery of care.

The specialty's reliance on different radiological imaging interpretations for diagnosing and treating injuries has given AI a chance to shine [4]. Studies have shown promising results regarding the ability of AI to analyze and process multiple images simultaneously, providing clinicians with faster and more efficient reports that contain fewer errors than those generated by conventional methods [5]. AI applications extend to perioperative planning for open reduction internal fixation and major joint replacements [6,7]. Before surgery, AI can be used to optimize patient and surgical factors. Patient factor optimization includes risk assessment and personalized guidance to decrease postoperative complications [8]. Surgical factor optimization includes implant choice, surgical approach choice, and virtual reality simulation, which allows surgeons to experience all possible scenarios before starting the operation [9].

Today, AI encompasses numerous subfields, with machine learning (ML) being the most promising for medical research. Deep learning represents a revolutionary approach in ML that can operate independently using multiple layers of artificial neurons to process information, mimicking the structure of the human brain [10-12]. For example, when examining hip radiographs, early layers might detect simple edges, middle layers identify bone structures, and deeper layers recognize complex patterns, such as fracture lines, all without being explicitly programmed to look for [13]. Deep learning achieves this function through convolutional neural networks (CNNs), which are a specialized type of deep learning designed specifically for analyzing images, surgical videos, electronic health records, and data from wearable sensors to detect patterns that are not visible to the human eye, making them ideal for orthopaedic applications [14,15].

**Figure 1:**
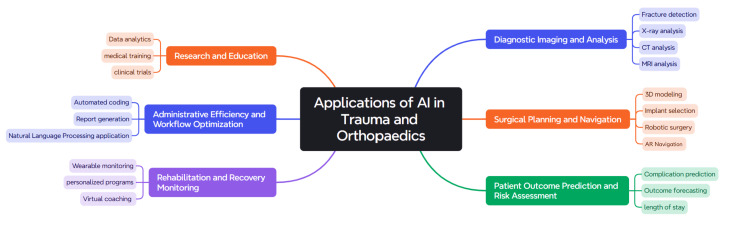
AI applications in trauma and orthopaedics This figure has been created using the Mapify application by author Ahmed Mohamed.

This narrative review aims to discuss the deep involvement of AI in many aspects of trauma and orthopaedics, as explained in Figure 1. We highlight the importance of involving human thoughts with AI in the early stages to keep AI diversions that might occur under control.

## Review

Historical evolution of AI in orthopaedics

The use of AI in orthopaedic practice has been developing for almost seven decades. The term "artificial intelligence" was introduced by John McCarthy at Dartmouth College in 1956, building on Alan Turing's earlier question of whether machines could think [16,17]. McCarthy and his team believed computers could be programmed to learn and demonstrate intelligence. However, practical applications in orthopaedics did not emerge until 1992 with the introduction of ROBODOC, the first robotic system specifically designed for orthopaedic surgery [18].

The early years of robotic orthopaedic surgery faced significant challenges. Initial systems suffered from prolonged operative times, increased blood loss, and higher infection rates [19]. The application of ML and training algorithms has helped overcome these challenges [20].

The period from 2000 to 2010 witnessed the beginning of AI invasion into healthcare domains [21]. This has occurred due to the convergence of three factors: exponential increases in computational power, widespread adoption of electronic medical records, and the development of sophisticated ML algorithms [22].

The current decade has witnessed AI achieving clinical maturity across multiple applications. Deep learning algorithms now match or exceed human performance in many diagnostic tasks, while integrated systems support the entire continuum of care [23]. The COVID-19 pandemic accelerated the adoption of AI, particularly in remote monitoring and telemedicine applications, demonstrating AI's value in maintaining care continuity during disruptions [24,25].

Diagnostic imaging and detection

**Figure 2:**
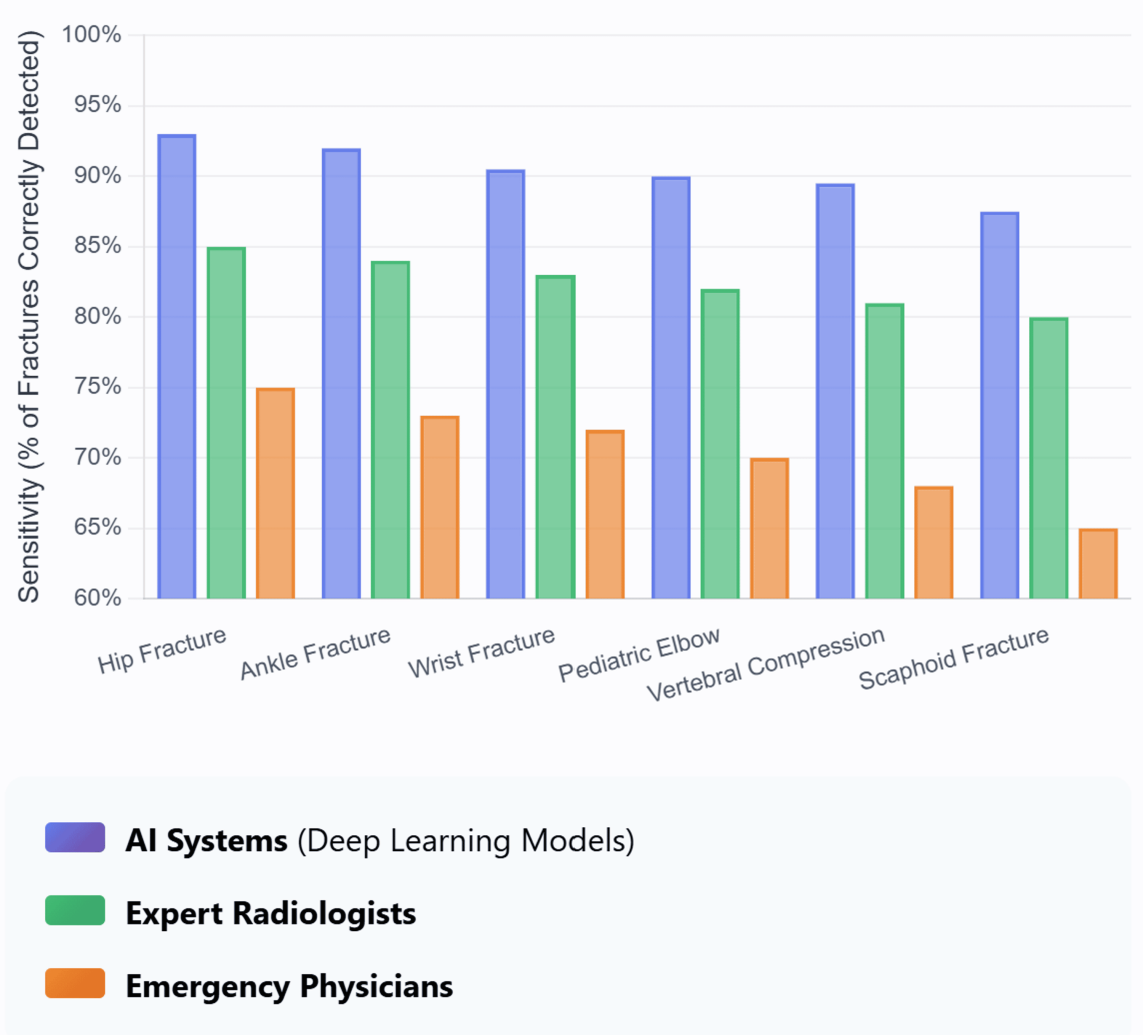
Diagnostic accuracy comparison: AI vs. human performance This figure has been created using the Claude website by author Ahmed Mohamed.

Diagnostic imaging is the cornerstone of orthopaedic practice. Fracture detection represents the most mature AI application in orthopaedics [26]. In Figure 2, we are explaining the high efficiency of AI in detecting different fracture types, achieving "88-95% accuracy as per Lindsey and colleagues [27]. The application of deep learning algorithms, particularly CNNs, has improved the ability to detect fractures and other pathological findings across all imaging modalities [28,29]. In Table 1, we discuss the high functionality of AI-assisted ML in detecting different types of fractures.

**Table 1:** Performance metrics of AI systems in common fracture detection tasks AUC (area under the curve) refers to the overall accuracy score. The value of this metric ranges from 0 to 1. An AUC of 1 indicates a perfect value (never wrong), 0.5 indicates an unreliable value, and 0.90-0.99 indicates excellent results. Dataset size refers to the number of X-rays used to test the AI. Validation type refers to how AI was tested; internal means all data were from the same hospital, and external means data were tested from different hospitals.

Fracture Type	Sensitivity (%)	Specificity (%)	AUC	Dataset Size	Validation Type	References
Hip Fracture	91–95	92–96	0.94–0.97	3,000–45,000	External	[30,31]
Wrist Fracture	88–93	89–94	0.91–0.95	2,500–15,000	Internal/External	[32]
Ankle Fracture	90–94	91–95	0.93–0.96	1,800–8,000	External	[33]
Vertebral Compression	87–92	88–93	0.90–0.94	2,000–12,000	Internal/External	[26]
Scaphoid Fracture	85–90	90–94	0.89–0.93	1,200–5,000	Internal	[34]
Pediatric Elbow	88–92	87–92	0.90–0.94	3,000–10,000	External	[35]

The clinical impact of these systems extends beyond simple detection of abnormalities. These deep learning models can classify fractures, evaluate fracture direction and angulation, and even suggest treatment strategies [36-38]. Integration with picture archiving and communication systems (PACS) has enabled many institutions to use AI for case triage and prioritization [39,40].

The scope of AI implementation in imaging interpretation includes computed tomography (CT) and magnetic resonance imaging (MRI) analysis [41]. Deep learning models have taken this function to a high level by analyzing complex fractures in CT and suggesting virtual reduction models, providing surgeons with ideas about potential approaches to optimal fixation strategies [42]. Deep learning models have a sensitivity of approximately 87% and a specificity of 89% in detecting and marking cuts where meniscal tears and anterior cruciate ligament tears are found in MRI without human intervention, exceeding expert radiologist performance [43].

Surgical planning and navigation

The complexity of modern orthopaedic surgery demands precise preoperative planning and intraoperative performance. AI has made perioperative planning precise by generating different models. 3D modelling represents a fundamental advance in surgical planning [44]. AI algorithms create patient-specific three-dimensional models through a deep analysis of CT and MRI [6]. These models enable surgeons to visualize the targeted pathology from any angle, plan precise surgical approaches, and reduce the likelihood of unexpected intraoperative occurrences [45].

The concept of a "digital twin" has emerged as a powerful AI application [46]. This involves creating virtual and dynamic replicas of the patient's anatomy and physiology. These models enable the simulation of various surgical approaches, patients' clinical responses to interventions, prediction of potential complications, and estimation of recovery outcomes [46,47]. This gives surgeons a chance to optimize their interventions before starting the operation to ensure the best possible outcome.

Implant selection is a growing challenge in trauma and orthopaedics [48]. Different manufacturers offer different types of implants for the same fractures. Choosing the right implant requires a deep analysis of the fracture pattern, patient anatomy, biomechanics, and bone quality. AI algorithms predict implant sizes with over 85% accuracy, reducing operative time and minimizing intraoperative adjustments [9,49]. In revision surgeries, it is sometimes very difficult to identify which implant has been used from radiographs. AI algorithms identify existing implants from radiographs with over 90% accuracy across multiple manufacturers, saving valuable operative time and reducing inventory waste [50].

Robot-assisted surgery represents the convergence of AI, computer vision, and precision mechanics [51]. Modern robotic systems employ AI for real-time bone tracking, soft-tissue balancing, and dynamic plan adjustment [52]. These systems achieve precise accuracy in millimetres in bone preparation and implant positioning. The use of robots shines even more in minimally invasive procedures when visualization is limited. In spine surgery, robot-assisted pedicle screw placement shows accuracy rates exceeding 95%, compared to 85-90% with free-hand fluoroscopy-guided methods [53]. Additionally, these systems reduce radiation exposure for both patients and surgical teams by minimizing fluoroscopy requirements [54].

Augmented reality (AR) navigation systems are an emerging surgical technology. This technique uses special headsets or glasses that overlay computer-generated images, including real-time 3D images of the patient's anatomy, implant template, and measurements, directly onto the surgeon's view of the operative field [55,56]. This technology enables the surgeon to focus on important structures without diverting their attention to different monitors [57]. This "see-through" guidance acts like a GPS for surgery, improving the accuracy of implant positioning, reducing radiation exposure, and potentially shortening operative time [58,59].

**Figure 3:**
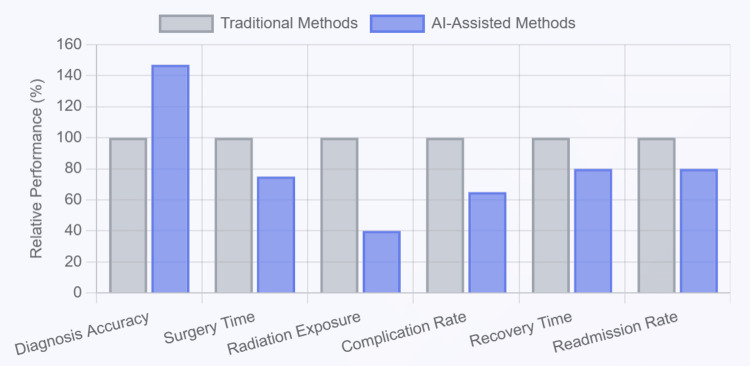
Clinical impact: AI vs. traditional methods This figure has been created using the Claude website by author Ahmed Mohamed.

Figure 3 illustrates the transformative impact of AI-assisted methods compared to traditional approaches.

Predictive analytics and risk assessment

AI has demonstrated high efficiency in predicting surgical outcomes, complication risks, and recovery trajectories [60]. ML has been trained to analyze patient-specific data, including demographics, comorbidities, laboratory values, imaging findings, and social determinants of health, to predict complications, length of stay, and forecast outcomes [61-63]. In Table 2, we examined the predictive capabilities of AI in identifying potential complications in routine orthopaedic surgeries. These predictions enable the development of proactive management strategies and appropriate resource allocation to enhance patient outcomes.

**Table 2:** Applications of predictive AI models in trauma and orthopaedics AUC: area under the curve; ACL: anterior cruciate ligament; BMI: body mass index

Clinical Application	Prediction Target	Model Performance (AUC)	Key Variables	Clinical Impact	References
Joint Arthroplasty	30-day readmission	0.78–0.85	Age, comorbidities, surgical time	20% reduction in readmissions	[64]
Hip Fracture	1-year mortality	0.82–0.89	Age, frailty index, albumin levels	Risk stratification for intensive intervention	[65]
Spine Surgery	Surgical site infection	0.75–0.84	BMI, diabetes, operative time	Targeted antibiotic prophylaxis	[1]
Sports Medicine	ACL re-rupture	0.77–0.83	Age, activity level, graft type	Personalized rehabilitation protocols	[66,67]
Trauma	Need for massive transfusion	0.85–0.91	Vital signs, injury severity, lab values	Early blood product preparation	[34]
Fracture Care	Nonunion risk	0.73–0.81	Fracture pattern, smoking, diabetes	Enhanced monitoring and intervention	[68]
Total Joint Arthroplasty	Periprosthetic joint infection risk	0.80–0.92	Prior infection, BMI, diabetes, operative time	Risk stratification for prophylaxis	[69]
Total Hip Arthroplasty	Hip dislocation likelihood	0.87	Postoperative radiograph features, cup angle, offset	Early identification of at-risk patients	[70]

Rehabilitation and recovery

Long-term outcomes after orthopaedic surgeries are determined by postsurgical rehabilitation programs. AI has transformed the way we guide and monitor recovery through the innovative application of wearable technology, virtual assistants, and intelligent monitoring systems.

Wearable monitoring uses sensors in devices such as smartwatches, activity trackers, and smart rings to continuously assess patient progress [71]. AI algorithms convert raw sensor data into clinically relevant metrics. Smart rings can predict acute fluctuations in postoperative pain after orthopaedic surgery [72]. Ramkumar and colleagues validated an ML-based remote monitoring platform for total knee arthroplasty patients that accurately tracks recovery metrics and identifies patients requiring intervention [73].

Virtual physical therapy assistant applications represent a paradigm shift in rehabilitation delivery. They guide patients through exercises using smartphone cameras and computer vision technology for movement analysis [74]. They ensure that exercises are performed correctly, provide real-time feedback on form, and adjust protocols dynamically based on progress. Virtual assistants achieve compliance rates exceeding 80%, compared to 50-60% with traditional home exercise programs [75]. This technology is especially valuable for patients in rural areas or those with transportation limitations. Moreover, they decrease the pressure on physiotherapists. During the COVID-19 pandemic, these systems proved essential for maintaining care continuity when in-person therapy was unavailable [76].

Administrative and documentation

Administrative jobs are a burden on healthcare providers. They consume valuable time that could be spent on patients. AI offers powerful solutions to streamline these essential but time-consuming tasks, improving efficiency while maintaining quality and compliance with regulations.

Automated coding uses natural language processing (NLP) to extract procedure codes and complications from operative notes and clinical documentation, with accuracy rates exceeding 90% [77,78]. This automation allows coding professionals to focus on complex cases requiring human expertise, while routine cases are processed automatically. This technology has reduced coding time by up to 50% while improving accuracy [79].

Report generation is a significant challenge, especially on busy clinical days. Large language models can generate discharge summaries, operative reports, and clinic notes in seconds rather than minutes or hours while maintaining the same quality as clinicians' reports [80]. Recent pilot studies have demonstrated that GPT-4 generates discharge summaries while reducing documentation time by 40% [81,82]. NLP is particularly important in the clinical setting. Physicians can dictate notes while AI structures the information appropriately through voice-to-text transformation with error rates below 2% for medical vocabulary [79].

Research and education

AI accelerates scientific discovery and enhances medical education in orthopaedics through innovative applications that transform research, training of future surgeons, and clinical data analysis.

The importance of AI in medical research begins with study selection and design [83]. ML algorithms can identify eligible patients and sample sizes through a deep analysis of health records and databases, increasing enrollment rates by up to 30% [84,85]. Moreover, recent AI models can predict patients who will most likely complete the trials, helping researchers optimize recruitment strategies and reduce dropout rates [86].

When it comes to writing and publishing, AI tools can perform full statistical analyses, generate tables and graphs, and suggest subheadings that the researcher can build the whole article on [87]. AI-powered applications can transform individual thoughts and simplify, paraphrase, or convert them into academic writing. This function is particularly valuable for non-English speakers who struggle with academic writing [88].

Medical training has been revolutionized by AI-powered simulators and educational tools. Virtual reality and simulation systems help trainees practice procedures in risk-free environments with unlimited repetitions [89]. AI provides personalized feedback based on performance, identifies areas needing improvement, and creates personalized training plans based on individual skill levels [90].

Challenges and limitations

Despite remarkable advances, significant challenges limit AI's full potential of AI in orthopaedics. Understanding these limitations is crucial for their successful implementation and continued development.

Ethical concerns regarding data security are a fundamental challenge in relying on AI. AI systems are trained using large amounts of patient data. The way these data were handled later is questionable [91]. Bias regarding the algorithms used to train AI is another challenge. Datasets used to train AI are often not diverse and represent only a single institution or a small group of people, which poses a challenge to the generalizability of these data outcomes [92]. The AI algorithms themselves are considered a "black box." Healthcare systems may be reluctant to employ AI systems whose decision-making processes cannot be clearly explained. Error analysis and system improvement in black box algorithms when failure occurs can be impossible, making their reliability poor [93].

Substituting traditional and well-established healthcare systems with new AI systems is another challenge. This will require hardware tools, software licensing, staff training, and workflow redesign, which can be prohibitive for smaller institutions or those in resource-limited settings [94].

Future directions

The effect of AI on trauma and orthopaedics is promising and will likely shape the field in the coming years. Federated learning (FL) is emerging as a solution to data privacy concerns. Instead of data centralization, FL allows data to be stored only on the user's device, which keeps the data local and maintains privacy [95].

The integration of multiple AI modalities into comprehensive clinical decision-making is revolutionary. Future systems will combine imaging analysis, outcome prediction, surgical planning, and rehabilitation monitoring into a unified platform supporting the entire continuum of care [96]. These integrated systems will provide consistent, evidence-based recommendations throughout the patient's journey.

Although quantum computing is still in its early stages, it has the potential to dramatically accelerate certain AI tasks. These new systems can analyze data and optimize surgical planning through unparalleled approaches that go beyond the scope of traditional computers [97].

The introduction of explainable AI makes it more transparent and trustworthy. New techniques that can visualize and explain the way of thinking and decision-making have been introduced to the market. This allows clinicians to detect any errors and redirect the systems to solve them, facilitating appropriate clinical integration [98]. Educational programs should be introduced to prepare surgeons to effectively utilize these tools while maintaining critical thinking when AI recommendations conflict with clinical judgment.

## Conclusions

The integration of AI into trauma and orthopaedic practice represents a paradigm shift in the diagnosis, treatment, and management of musculoskeletal conditions. AI systems are guiding the patient management journey from diagnosis to personalized rehabilitation protocols. Success requires collaborative efforts involving clinicians, data scientists, and policymakers to overcome the current challenges. The development of robust validation frameworks, standardized performance metrics, and transparent reporting guidelines is essential for building trust and ensuring safe deployment.

Looking forward, the future of trauma and orthopaedics will be characterized by seamless integration of AI technologies throughout the entire care pathway. The key to successful integration lies in maintaining a patient-centred approach, ensuring equitable access to these technologies, and preserving the essential human elements of compassion, creativity, and clinical wisdom that define excellent medical care. As we advance into this new era, research, continuous monitoring, and careful attention to ethical considerations will be crucial as AI becomes more involved in the field. The goal is not to replace human expertise but to augment it, creating a synergy between human judgment and machine precision that delivers high levels of care to all patients.

## References

[1] Panchmatia JR, Visenio MR, Panch T (2018). The role of artificial intelligence in orthopaedic surgery. Br J Hosp Med (Lond).

[2] Gitto S, Serpi F, Albano D, Risoleo G, Fusco S, Messina C, Sconfienza LM (2024). AI applications in musculoskeletal imaging: a narrative review. Eur Radiol Exp.

[3] Nguyen A, Lee P, Rodriguez EK, Chahal K, Freedman BR, Nazarian A (2025). Addressing the growing burden of musculoskeletal diseases in the ageing US population: challenges and innovations. Lancet Healthy Longev.

[4] Bhandari A (2024). Revolutionizing radiology with artificial intelligence. Cureus.

[5] Zhong Z, Xie X (2024). Clinical applications of generative artificial intelligence in radiology: image translation, synthesis, and text generation. BJR Artif intell.

[6] Song J, Wang GC, Wang SC, He CR, Zhang YZ, Chen X, Su JC (2025). Artificial intelligence in orthopedics: fundamentals, current applications, and future perspectives. Mil Med Res.

[7] Han F, Huang X, Wang X (2025). Artificial intelligence in orthopedic surgery: current applications, challenges, and future directions. MedComm (2020).

[8] Hassan AM, Rajesh A, Asaad M, Nelson JA, Coert JH, Mehrara BJ, Butler CE (2023). Artificial intelligence and machine learning in prediction of surgical complications: current state, applications, and implications. Am Surg.

[9] Shah AK, Lavu MS, Hecht CJ 2nd, Burkhart RJ, Kamath AF (2023). Understanding the use of artificial intelligence for implant analysis in total joint arthroplasty: a systematic review. Arthroplasty.

[10] Sarker IH (2021). Deep learning: a comprehensive overview on techniques, taxonomy, applications and research directions. SN Comput Sci.

[11] Tieu A, Kroen E, Kadish Y (2024). The role of artificial intelligence in the identification and evaluation of bone fractures. Bioengineering (Basel).

[12] Yamamoto N, Sukegawa S, Kitamura A (2020). Deep learning for osteoporosis classification using hip radiographs and patient clinical covariates. Biomolecules.

[13] Ibrahim R, Shafiq MO (2023). Explainable convolutional neural networks: a taxonomy, review, and future directions. ACM Comput Surv.

[14] Kourounis G, Elmahmudi AA, Thomson B, Hunter J, Ugail H, Wilson C (2023). Computer image analysis with artificial intelligence: a practical introduction to convolutional neural networks for medical professionals. Postgrad Med J.

[15] Mienye ID, Swart TG, Obaido G, Ilono P, Jordan M (2025). Deep convolutional neural networks in medical image analysis: a review. Information.

[16] McCarthy J, Rochester N, Minsky M, Shannon C (2006). A proposal for the Dartmouth summer research project on artificial intelligence, August 31, 1955. AI Mag.

[17] Farhadi F, Barnes MR, Sugito HR, Sin JM, Henderson ER, Levy JJ (2022). Applications of artificial intelligence in orthopaedic surgery. Front Med Technol.

[18] Lang JE, Mannava S, Floyd AJ (2011). Robotic systems in orthopaedic surgery. J Bone Joint Surg Br.

[19] Beyaz S (2020). A brief history of artificial intelligence and robotic surgery in orthopedics & traumatology and future expectations. Jt Dis Relat Surg.

[20] Maffulli N, Rodriguez HC, Stone IW (2020). Artificial intelligence and machine learning in orthopedic surgery: a systematic review protocol. J Orthop Surg Res.

[21] Hirani R, Noruzi K, Khuram H (2024). Artificial intelligence and healthcare: a journey through history, present innovations, and future possibilities. Life (Basel).

[22] Karalis VD (2024). The integration of artificial intelligence into clinical practice. Appl Biosci.

[23] Iqbal J, Cortés Jaimes DC, Makineni P (2023). Reimagining healthcare: unleashing the power of artificial intelligence in medicine. Cureus.

[24] Bokolo Anthony Jnr (2020). Use of telemedicine and virtual care for remote treatment in response to COVID-19 pandemic. J Med Syst.

[25] Burrell DN (2023). Dynamic evaluation approaches to telehealth technologies and artificial intelligence (AI) telemedicine applications in healthcare and biotechnology organizations. Merits.

[26] Boginskis V, Sauka J, Beikmane D, Cernavska I, Zadoroznijs S (2023). Artificial intelligence effectivity in fracture detection. Med Perspekt.

[27] Lindsey R, Daluiski A, Chopra S (2018). Deep neural network improves fracture detection by clinicians. Proc Natl Acad Sci U S A.

[28] Alkhatib AJ, Alharoun M, Alzoubi A (2024). A deep learning framework for timely bone fracture detection and prevention. Information Sci Appl.

[29] Kutbi M (2024). Artificial intelligence-based applications for bone fracture detection using medical images: a systematic review. Diagnostics (Basel).

[30] Urakawa T, Tanaka Y, Goto S, Matsuzawa H, Watanabe K, Endo N (2019). Detecting intertrochanteric hip fractures with orthopedist-level accuracy using a deep convolutional neural network. Skeletal Radiol.

[31] Cheng CT, Ho TY, Lee TY (2019). Application of a deep learning algorithm for detection and visualization of hip fractures on plain pelvic radiographs. Eur Radiol.

[32] Kim DH, MacKinnon T (2018). Artificial intelligence in fracture detection: transfer learning from deep convolutional neural networks. Clin Radiol.

[33] SooHoo NF, Krenek L, Eagan MJ, Gurbani B, Ko CY, Zingmond DS (2009). Complication rates following open reduction and internal fixation of ankle fractures. J Bone Joint Surg Am.

[34] Langerhuizen DW, Janssen SJ, Mallee WH (2019). What are the applications and limitations of artificial intelligence for fracture detection and classification in orthopaedic trauma imaging? A systematic review. Clin Orthop Relat Res.

[35] Choi JW, Cho YJ, Lee S (2020). Using a dual-input convolutional neural network for automated detection of pediatric supracondylar fracture on conventional radiography. Invest Radiol.

[36] Anttila TT, Karjalainen TV, Mäkelä TO, Waris EM, Lindfors NC, Leminen MM, Ryhänen JO (2023). Detecting distal radius fractures using a segmentation-based deep learning model. J Digit Imaging.

[37] Kekatpure A, Kekatpure A, Deshpande S, Srivastava S (2024). Development of a diagnostic support system for distal humerus fracture using artificial intelligence. Int Orthop.

[38] Xie Y, Li X, Chen F, Wen R, Jing Y, Liu C, Wang J (2024). Artificial intelligence diagnostic model for multi-site fracture X-ray images of extremities based on deep convolutional neural networks. Quant Imaging Med Surg.

[39] Theriault-Lauzier P, Cobin D, Tastet O (2024). A responsible framework for applying artificial intelligence on medical images and signals at the point of care: the PACS-AI platform. Can J Cardiol.

[40] Pérez-Sanpablo AI, Quinzaños-Fresnedo J, Gutiérrez-Martínez J, Lozano-Rodríguez IG, Roldan-Valadez E (2025). Transforming medical imaging: the role of artificial intelligence integration in PACS for enhanced diagnostic accuracy and workflow efficiency. Curr Med Imaging.

[41] Miller DD, Brown EW (2019). How cognitive machines can augment medical imaging. AJR Am J Roentgenol.

[42] Dankelman LH, Schilstra S, IJpma FF (2023). Artificial intelligence fracture recognition on computed tomography: review of literature and recommendations. Eur J Trauma Emerg Surg.

[43] Behr J, Nich C, D'Assignies G (2025). Deep learning-assisted detection of meniscus and anterior cruciate ligament combined tears in adult knee magnetic resonance imaging: a crossover study with arthroscopy correlation. Int Orthop.

[44] Galvez M, Asahi T, Baar A (2018). Use of three-dimensional printing in orthopaedic surgical planning. J Am Acad Orthop Surg Glob Res Rev.

[45] Isikay I, Cekic E, Baylarov B, Tunc O, Hanalioglu S (2024). Narrative review of patient-specific 3D visualization and reality technologies in skull base neurosurgery: enhancements in surgical training, planning, and navigation. Front Surg.

[46] Dean MC, Oeding JF, Diniz P, Seil R, Samuelsson K (2024). Leveraging digital twins for improved orthopaedic evaluation and treatment. J Exp Orthop.

[47] Vallée A (2023). Digital twin for healthcare systems. Front Digit Health.

[48] Wähnert D, Greiner J, Brianza S, Kaltschmidt C, Vordemvenne T, Kaltschmidt B (2021). Strategies to improve bone healing: innovative surgical implants meet nano-/micro-topography of bone scaffolds. Biomedicines.

[49] Yu Y, Cho YJ, Park S, Kim YH, Goh TS (2024). Development of an artificial intelligence model for predicting implant size in total knee arthroplasty using simple X-ray images. J Orthop Surg Res.

[50] Tiwari A, Yadav AK, Akshay KS, Bagaria V (2023). Evaluation of machine learning models to identify hip arthroplasty implants using transfer learning algorithms. J Clin Orthop Trauma.

[51] Khaohoen A, Powcharoen W, Sornsuwan T, Chaijareenont P, Rungsiyakull C, Rungsiyakull P (2024). Accuracy of implant placement with computer-aided static, dynamic, and robot-assisted surgery: a systematic review and meta-analysis of clinical trials. BMC Oral Health.

[52] Ng MS, Loke RW, Tan MK, Ng YH, Liau ZQ (2025). Novel artificial intelligence algorithm for soft tissue balancing and bone cuts in robotic total knee arthroplasty improves accuracy and surgical duration. Arthroplasty.

[53] Aurouer N, Guerin P, Cogniet A, Gangnet N, Pedram M, Piechaud PT, Mangione P (2024). Pedicle screw placement accuracy in robot-assisted versus image-guided freehand surgery of thoraco-lumbar spine (ROBARTHRODESE): study protocol for a single-centre randomized controlled trial. Trials.

[54] Saeedi-Hosseiny MS, Rothenberg HG, Jadallah AL, Khaja U, Karimov A, Abedin-Nasab MH (2025). The impact of robotic surgery on reducing radiation exposure in orthopedic trauma: a meta-analysis. J Robot Surg.

[55] Edström E, Burström G, Omar A (2020). Augmented reality surgical navigation in spine surgery to minimize staff radiation exposure. Spine (Phila Pa 1976).

[56] Avrumova F, Lebl DR (2022). Augmented reality for minimally invasive spinal surgery. Front Surg.

[57] Neri A, Penza V, Baldini C, Mattos LS (2025). Surgical augmented reality registration methods: a review from traditional to deep learning approaches. Comput Med Imaging Graph.

[58] Chen F, Cui X, Han B, Liu J, Zhang X, Liao H (2021). Augmented reality navigation for minimally invasive knee surgery using enhanced arthroscopy. Comput Methods Programs Biomed.

[59] Hersh A, Mahapatra S, Weber-Levine C (2021). Augmented reality in spine surgery: a narrative review. HSS J.

[60] Huffman N, Pasqualini I, Khan ST (2024). Enabling personalized medicine in orthopaedic surgery through artificial intelligence: a critical analysis review. JBJS Rev.

[61] Li YY, Wang JJ, Huang SH, Kuo CL, Chen JY, Liu CF, Chu CC (2022). Implementation of a machine learning application in preoperative risk assessment for hip repair surgery. BMC Anesthesiol.

[62] Bihorac A, Ozrazgat-Baslanti T, Ebadi A (2019). MySurgeryRisk: development and validation of a machine-learning risk algorithm for major complications and death after surgery. Ann Surg.

[63] Girod MM, Saniei S, Ulrich MN, Bukowiec LG, Mulford KL, Taunton MJ, Wyles CC (2025). Artificial intelligence in the diagnosis and prognostication of the musculoskeletal patient. HSS J.

[64] Harris AH, Kuo AC, Weng Y, Trickey AW, Bowe T, Giori NJ (2019). Can machine learning methods produce accurate and easy-to-use prediction models of 30-day complications and mortality after knee or hip arthroplasty?. Clin Orthop Relat Res.

[65] Krogue J, Geiger E, Zaid M (2019). Automatic Hip Fracture Identification and Functional Subclassification with Deep Learning.

[66] Bien N, Rajpurkar P, Ball RL (2018). Deep-learning-assisted diagnosis for knee magnetic resonance imaging: development and retrospective validation of MRNet. PLoS Med.

[67] Liu F, Guan B, Zhou Z (2019). Fully automated diagnosis of anterior cruciate ligament tears on knee MR images by using deep learning. Radiol Artif Intell.

[68] Kalmet PH, Sanduleanu S, Primakov S (2020). Deep learning in fracture detection: a narrative review. Acta Orthop.

[69] Anderson MB, Curtin K, Wong J, Pelt CE, Peters CL, Gililland JM (2017). Familial clustering identified in periprosthetic joint infection following primary total joint arthroplasty: a population-based cohort study. J Bone Joint Surg Am.

[70] Rouzrokh P, Ramazanian T, Wyles CC (2021). Deep learning artificial intelligence model for assessment of hip dislocation risk following primary total hip arthroplasty from postoperative radiographs. J Arthroplasty.

[71] Adans-Dester C, Hankov N, O'Brien A (2020). Enabling precision rehabilitation interventions using wearable sensors and machine learning to track motor recovery. NPJ Digit Med.

[72] Morimoto M, Nawari A, Savic R, Marmor M (2024). Exploring the potential of a smart ring to predict postoperative pain outcomes in orthopedic surgery patients. Sensors (Basel).

[73] Ramkumar PN, Haeberle HS, Ramanathan D (2019). Remote patient monitoring using mobile health for total knee arthroplasty: validation of a wearable and machine learning-based surveillance platform. J Arthroplasty.

[74] Dutta S, Ambade R, Wankhade D, Agrawal P (2024). Rehabilitation techniques before and after total knee arthroplasty for a better quality of life. Cureus.

[75] Oginni J, Otinwa G, Gao Z (2024). Physical impact of traditional and virtual physical exercise programs on health outcomes among corporate employees. J Clin Med.

[76] Manes MR, Burnfield JM, Boersma K (2024). Virtual rehabilitation and COVID-19: varied adoption and satisfaction among patients and providers participating in a multi-site survey study. Inquiry.

[77] Venkatesh KP, Raza MM, Kvedar JC (2023). Automating the overburdened clinical coding system: challenges and next steps. NPJ Digit Med.

[78] Boonstra MJ, Weissenbacher D, Moore JH, Gonzalez-Hernandez G, Asselbergs FW (2024). Artificial intelligence: revolutionizing cardiology with large language models. Eur Heart J.

[79] Bai E, Luo X, Zhang Z, Adelgais K, Ali H, Finkelstein J, Kutzin J (2025). Assessment and integration of large language models for automated electronic health record documentation in emergency medical services. J Med Syst.

[80] Zernikow J, Grassow L, Gröschel J, Henrion P, Wetzel PJ, Spethmann S (2023). Clinical application of large language models: does ChatGPT replace medical report formulation? An experience report (Article in German). Inn Med (Heidelb).

[81] Tung JY, Gill SR, Sng GG (2024). Comparison of the quality of discharge letters written by large language models and junior clinicians: single-blinded study. J Med Internet Res.

[82] Dubinski D, Won SY, Trnovec S (2024). Leveraging artificial intelligence in neurosurgery-unveiling ChatGPT for neurosurgical discharge summaries and operative reports. Acta Neurochir (Wien).

[83] Oettl FC, Zsidai B, Oeding JF, Hirschmann MT, Feldt R, Tischer T, Samuelsson K (2025). Beyond traditional orthopaedic data analysis: AI, multimodal models and continuous monitoring. Knee Surg Sports Traumatol Arthrosc.

[84] Nashwan AJ, Hani SB (2023). Transforming cancer clinical trials: the integral role of artificial intelligence in electronic health records for efficient patient recruitment. Contemp Clin Trials Commun.

[85] Schwager E, Jansson K, Rahman A (2021). Utilizing machine learning to improve clinical trial design for acute respiratory distress syndrome. NPJ Digit Med.

[86] Anuyah S, Nyavor H, Singh M (2024). Advancing clinical trial outcomes using deep learning and predictive modelling: bridging precision medicine and patient-centered care. World J Adv Res Rev.

[87] Alammar A, Abdel-Reheem Amin E (2023). EFL students' perception of using AI paraphrasing tools in English language research projects. Arab World Engl J.

[88] Jamshed M, Sarfaraj M, Ahmed A, Warda W (2024). The impact of ChatGPT on English language learners' writing skills. Int J Interact Mob Technol.

[89] Ali M (2025). The role of AI in reshaping medical education: opportunities and challenges. Clin Teach.

[90] Sriram A, Ramachandran K, Krishnamoorthy S (2025). Artificial intelligence in medical education: transforming learning and practice. Cureus.

[91] Mennella C, Maniscalco U, De Pietro G, Esposito M (2024). Ethical and regulatory challenges of AI technologies in healthcare: a narrative review. Heliyon.

[92] Norori N, Hu Q, Aellen FM, Faraci FD, Tzovara A (2021). Addressing bias in big data and AI for health care: a call for open science. Patterns (N Y).

[93] Weiner EB, Dankwa-Mullan I, Nelson WA, Hassanpour S (2025). Ethical challenges and evolving strategies in the integration of artificial intelligence into clinical practice. PLOS Digit Health.

[94] Maleki Varnosfaderani S, Forouzanfar M (2024). The role of AI in hospitals and clinics: transforming healthcare in the 21st century. Bioengineering (Basel).

[95] Shahzad H, Veliky C, Le H, Qureshi S, Phillips FM, Javidan Y, Khan SN (2024). Preserving privacy in big data research: the role of federated learning in spine surgery. Eur Spine J.

[96] Khosravi M, Zare Z, Mojtabaeian SM, Izadi R (2024). Artificial intelligence and decision-making in healthcare: a thematic analysis of a systematic review of reviews. Health Serv Res Manag Epidemiol.

[97] Ali H (2023). Quantum computing and AI in healthcare: accelerating complex biological simulations, genomic data processing, and drug discovery innovations. World J Adv Res Rev.

[98] Metta C, Beretta A, Pellungrini R, Rinzivillo S, Giannotti F (2024). Towards transparent healthcare: advancing local explanation methods in explainable artificial intelligence. Bioengineering (Basel).

